# Supplementary material for: Quantum spin liquid signatures in monolayer 1T-NbSe2
Source: Nat Commun. 2024 Mar 15;15:2336. doi: 10.1038/s41467-024-46612-1 (PMC10940636; doi:10.1038/s41467-024-46612-1)
Supplement: Supplementary file 1 — Supplementary Information [file 41467_2024_46612_MOESM1_ESM.pdf]

# Supplementary Information

# Quantum spin liquid signatures in monolayer 1T-$NbSe_2$

Quanzhen Zhang[1,#], Wen-Yu He[2,#], Yu Zhang[1,3,#,*],Yaoyao Chen[1,#], Liangguang Jia[1], Yanhui Hou[1], Hongyan Ji[1], Huixia Yang[1], Teng Zhang[1], Liwei Liu[1], Hong-Jun Gao[4], Thomas A Jung[5], and Yeliang Wang[1,6,*]

[1]School of Integrated Circuits and Electronics, MIIT Key Laboratory for Low-Dimensional Quantum Structure and Devices, Beijing Institute of Technology, Beijing 100081, China.

[2]School of Physical Science and Technology, ShanghaiTech University, Shanghai 201210, China.

[3]Advanced Research Institute of Multidisciplinary Sciences, Beijing Institute of Technology, Beijing 100081, China.

[4]Institute of Physics, Chinese Academy of Sciences, Beijing 100190, China.

[5]Laboratory for X-ray Nanoscience and Technologies, Paul Scherrer Institut (PSI), 5232 Villigen, Switzerland.

[6]Yangtze Delta Region Academy, Beijing Institute of Technology, Jiaxing, Zhejiang 314000, China.

[*]Correspondence and requests for materials should be addressed to Yu Zhang (e-mail: yzhang@bit.edu.cn) and Yeliang Wang (e-mail: yeliang.wang@bit.edu.cn).

[#]Q.Z.Z., W.Y.H., Y.Z., and Y.Y.C. contributed equally to this work.

## Supplementary Notes 1-4

Supplementary Note 1: Rule out the possibility of the graphene substrate and the tip effect as the origin of the incommensurate electronic modulations.

Supplementary Note 2: Spatial variations of the resonance peak near the UHB edge.

Supplementary Note 3: Spinon Kondo effect

Supplementary Note 4: Differences in electronic properties among 1T-$NbSe_2$, 1T-$TaS_2$, and 1T-$TaSe_2$.

**Supplementary Figs. 1-20**

Supplementary Fig. 1 | Large-scale topographic STM image.

Supplementary Fig. 2 | Atomic and SOD structures of monolayer 1T-$NbSe_2$.

Supplementary Fig. 3 | Atomic structure of 1H-$NbSe_2$.

Supplementary Fig. 4 | STM image and STS spectra of bilayer 1T-$NbSe_2$.

Supplementary Fig. 5 | STM image and orbital textures in monolayer 1T-$NbSe_2$.

Supplementary Fig. 6 | Atomic-resolution STM image of monolayer 1T-$NbSe_2$ o.

Supplementary Fig. 7 | Movement of monolayer 1T-$NbSe_2$ on BLG.

Supplementary Fig. 8 | Incommensurate electronic modulations of monolayer 1T-$NbSe_2$.

Supplementary Fig. 9 | Electronic properties of monolayer 1H-$NbSe_2$.

Supplementary Fig. 10 | FFT images of the STS maps.

Supplementary Fig. 11 | MnPc molecules with two topographic configurations.

Supplementary Fig. 12 | Spatially resolved intensities for MnPc-top.

Supplementary Fig. 13 | Spatially resolved intensities for MnPc-hollow.

Supplementary Fig. 14 | Atomic and electronic structures of ZnPc on 1T-$NbSe_2$.

Supplementary Fig. 15 | Spatially resolved charge intensities for ZnPc-top.

Supplementary Fig. 16 | Spatially resolved charge intensities for ZnPc-hollow.

Supplementary Fig. 17 | The DOS in the pristine QSL and at the position of the adsorbate acting like a Kondo-impurity.

Supplementary Fig. 18 | The DOS in the pristine QSL and at the position of the adsorbate acting like a Kondo-impurity.

Supplementary Fig. 19 | The emergence of a pair of band edge resonance peaks in the local electronic DOS of the local magnetic impurity.

Supplementary Fig. 20 | Spatially resolved STS spectra of monolayer 1T-$NbSe_2$.

**Supplementary Table 1**

Supplementary Table 1 | Summarization of discrepancies among 1T-$NbSe_2$, 1T-$TaS_2$, and 1T-$TaSe_2$.

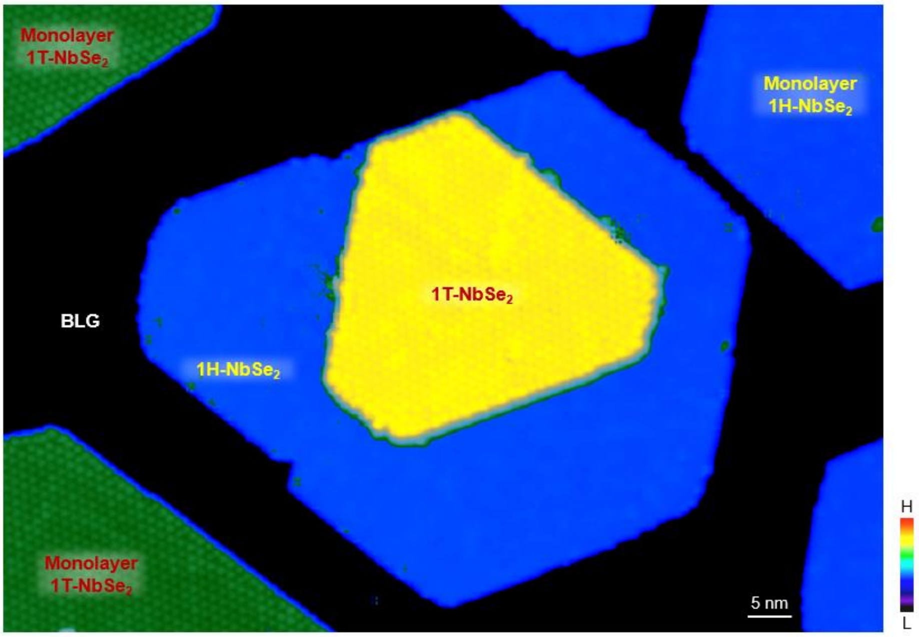


**Supplementary Fig. 1 | Large-scale topographic STM image of monolayer 1H-$NbSe_2$, 1T-$NbSe_2$, and their vertical heterostructure on BLG/SiC(0001) substrate ($V_b$ = -1.5 V, $I_t$ = 10 pA).**

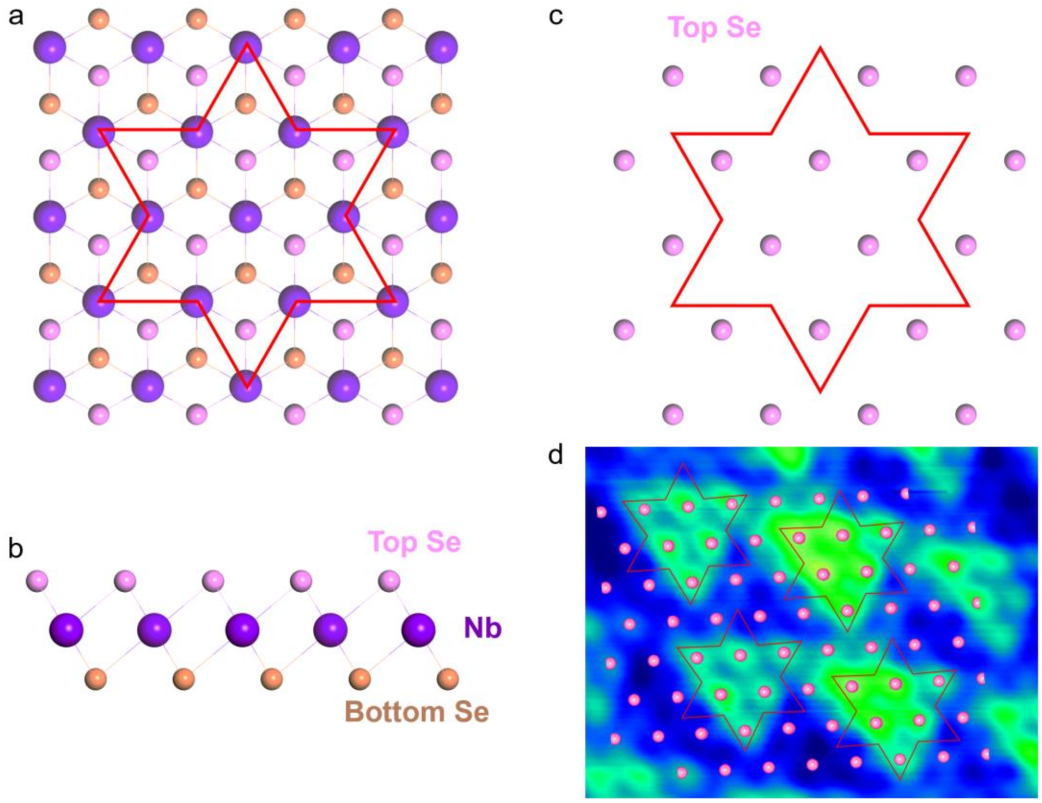


**Supplementary Fig. 2 | Atomic and SOD structures of monolayer 1T-NbSe$_2$. a,b,** Top and side views of atomic and SOD structures of monolayer 1T-NbSe$_2$. Nb atoms are shown in purple, and top/bottom Se atoms are exhibited in pink/orange. **c,** Top-view atomic structure of top Se atoms in monolayer 1T-NbSe$_2$, exhibiting a triangular object in each SOD motif. **d,** Atomic-resolution STM image of monolayer 1T-NbSe$_2$. Since STM images mainly reflect the information about the topmost Se layer, each SOD motif is imaged as a triangular object.

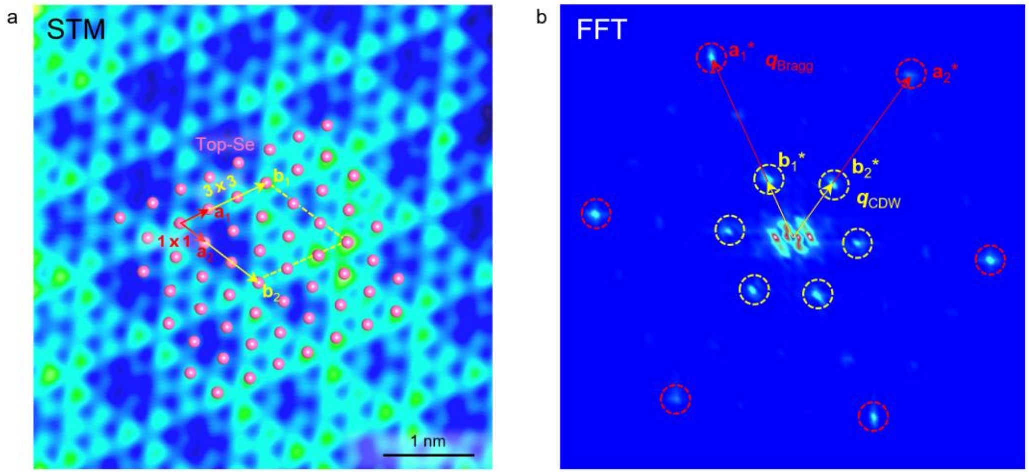


**Supplementary Fig. 3 | Atomic structure of 1H-NbSe2. a,** Atomic resolution STM image of monolayer 1H-$NbSe_2$ on BLG/SiC(0001) substrate recorded at the sample bias of -0.1 V. **b,** The corresponding FFT image of the atomic STM image of monolayer 1H-$NbSe_2$. The six bright spots enclosed by the dashed red circles and the six bright spots enclosed by the dashed yellow circles indicate the atomic and CDW wavevectors of monolayer 1H-$NbSe_2$ with 1 × 1 and 3 × 3 patterns, respectively. The basis vectors of the atomic and CDW pattern are indicated as $a_1^*/a_2^*$ and $b_1^*/b_2^*$, respectively.

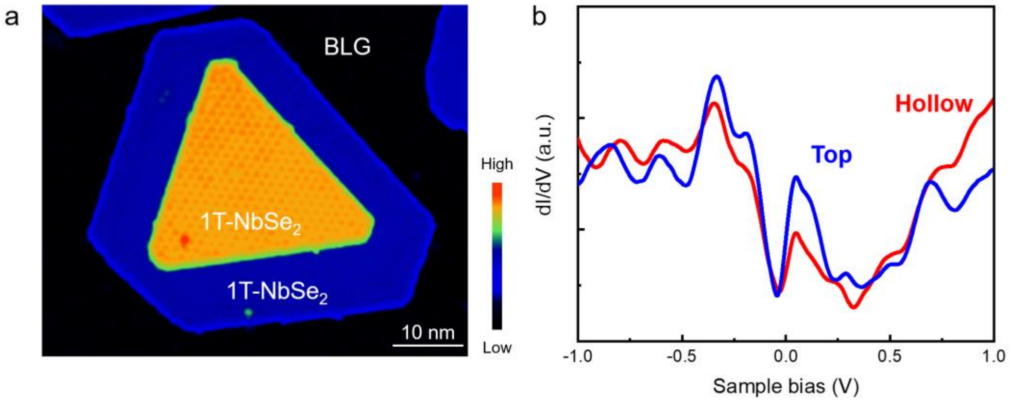


**Supplementary Fig. 4 | STM image and STS spectra of bilayer 1T-NbSe2. a,** Large-scale STM topographic image of bilayer 1T-$NbSe_2$ on BLG/SiC(0001) substrate ($V_b$ = -1.5 V, $I_t$ = 10 pA). b, Typical STS spectra of bilayer 1T-$NbSe_2$ recorded on the top and hollow sites of CDW pattern in the topmost 1T-$NbSe_2$ layer, suggesting a metallic state.

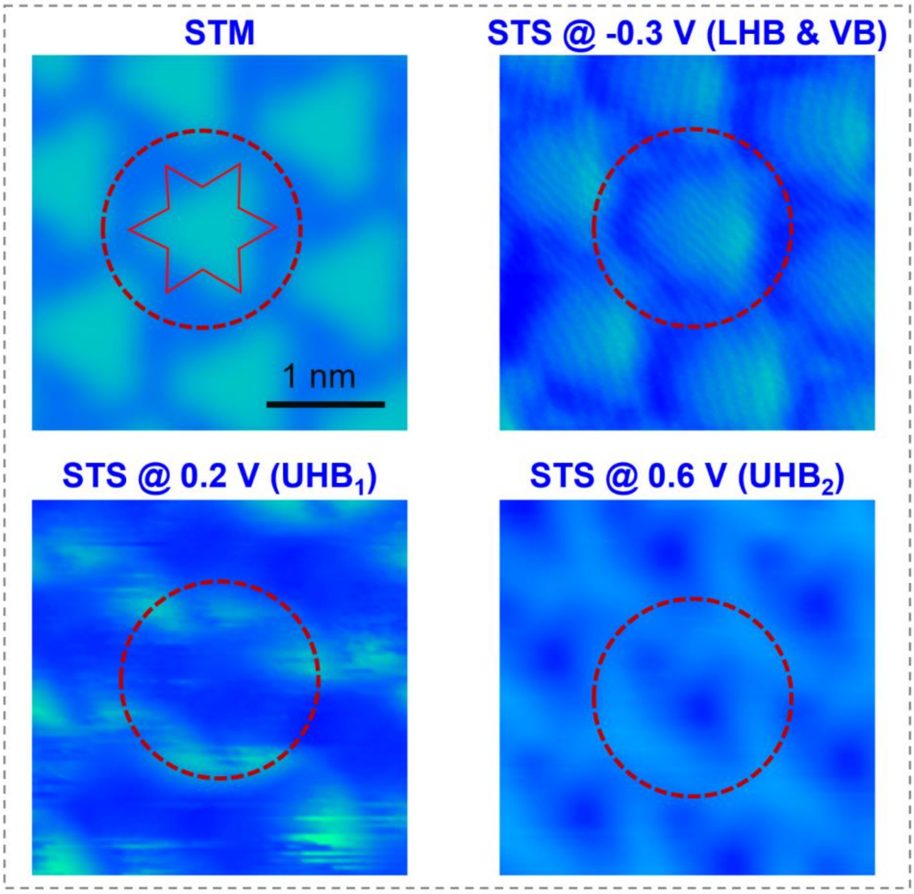


**Supplementary Fig. 5 | STM image and orbital textures of LHB & VB, $UHB_1$ and $UHB_2$ in monolayer 1T-$NbSe_2$.**

## Supplementary Note 1: Rule out the possibility of the graphene substrate and the tip effect as the origin of the incommensurate electronic modulations.

Firstly, we rule out the possibility of the graphene substrate as the origin of the incommensurate electronic modulations. The wavelength of the moiré pattern constructed by 1T-$NbSe_2$ and graphene can be obtained by

$$\lambda = \frac{(1+\delta)a_G}{\sqrt{2(1+\delta)(1-\cos\phi)+\delta^2}}$$

where $a_G \approx 0.246\ \text{nm}$ is the lattice constant of graphene, $a_{NbSe_2} \approx 0.346\ \text{nm}$ is the lattice constant of 1T-$NbSe_2$, and the lattice mismatch between 1T-$NbSe_2$ and graphene $\delta = (a_{NbSe_2} - a_G)/a_G \approx 0.407$. The relative rotation angle $\phi$ between 1T-$NbSe_2$ and graphene lattices shown in Fig. 1b is about 28°, according to the atomic-resolution STM images shown in Supplementary Fig. 6. In such a case, the wavelength of the moiré pattern $\lambda \approx 0.491\ \text{nm}$, which is quite inconsistent with the observed incommensurate electronic-state modulations. Moreover, we develop an in-situ STM manipulation technique to precisely control the relative angle between 1T-$NbSe_2$ and graphene lattices, as shown in Supplementary Fig. 7. We find out that the observed incommensurate electronic modulations are invariable under different relative angles (Supplementary Fig. 8). Therefore, we can rule out the possibility of the moiré pattern as the origin of the incommensurate electronic-state modulations.

Secondly, we rule out the possibility of the tip effect as the origin of the incommensurate electronic modulations. On the one hand, during our measurements, the incommensurate electronic modulations only appear when the recorded energies are close to the Hubbard band edges of monolayer 1T-$NbSe_2$. On the other hand, we carry out the STS maps of monolayer 1H-$NbSe_2$ (a CDW metal) on graphene under the same tip conditions. We find out that the STS map recorded at the energy of -0.2 eV exhibits no additional electronic modulation, as shown in Supplementary Fig. 9. These

phenomena highlight that the incommensurate electronic modulations are the intrinsic electronic properties of monolayer 1T-$NbSe_2$ near the Hubbard band edges, helping us rule out the possibility of the tip effect as the origin of the incommensurate electronic modulations.

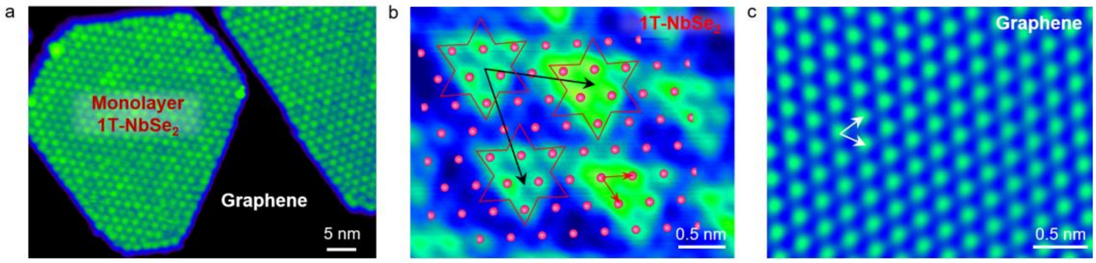


**Supplementary Fig. 6 | Atomic-resolution STM image of monolayer 1T-$NbSe_2$ on graphene. a,** Large-scale STM topographic image of monolayer 1T-$NbSe_2$ on graphene. **b,** Atomic-resolution STM image of 1T-$NbSe_2$. In each SOD motif, the top Se atoms dominate the STM images as a triangular bright protrusion. The basis vectors of $1 \times 1$ atomic lattices and $(\sqrt{13} \times \sqrt{13})$ $R$ 13.9° CDW lattices are marked by red and black arrows, respectively. **c,** Atomic-resolution STM image of graphene. The basis vectors of $1 \times 1$ atomic lattices are marked by white arrows.

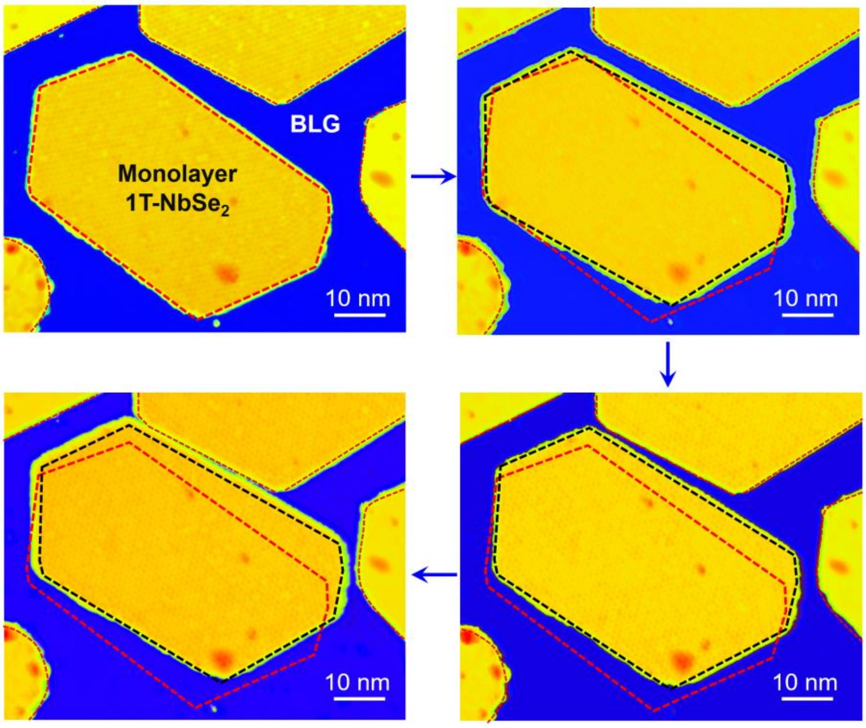


**Supplementary Fig. 7 | Movement of monolayer 1T-$NbSe_2$ on BLG via an in-situ STM manipulation technique.** The atomic orientations between monolayer 1T-$NbSe_2$ and BLG can be arbitrary during our manipulation, which can be captured by STM images. Here the red dotted outline donates the initial location of the central monolayer 1T-$NbSe_2$, and the black dotted outline donates its location after the manipulation.

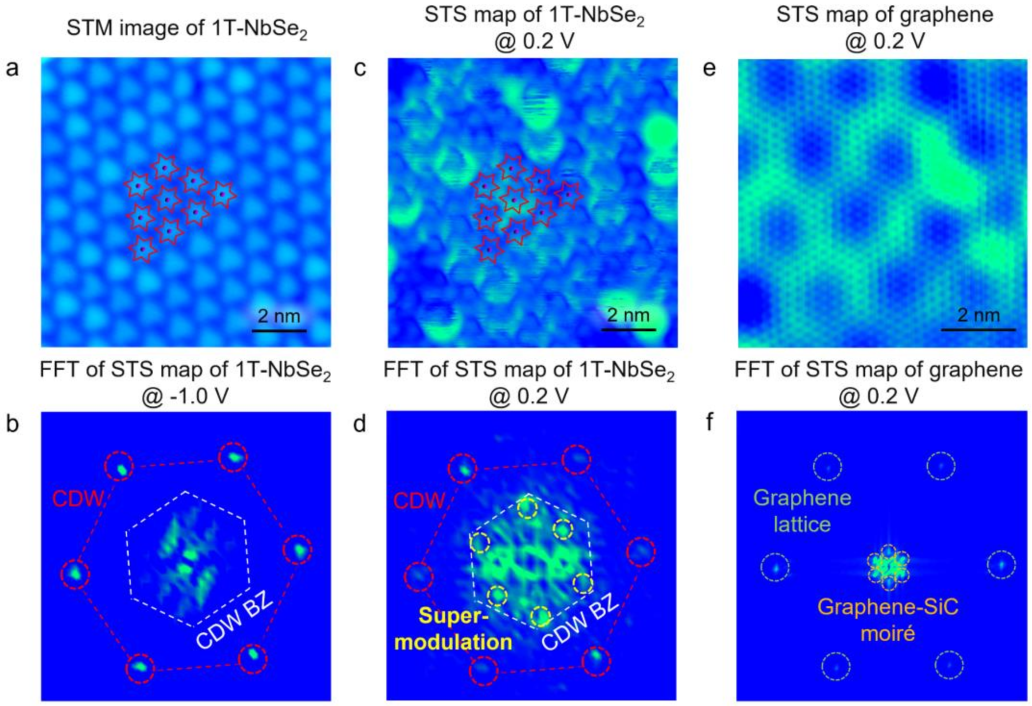


**Supplementary Fig. 8 | Incommensurate electronic modulations of monolayer 1T-$NbSe_2$. a,** STM image of another monolayer 1T-$NbSe_2$ recorded at the sample bias of -1 V. **b,** FFT image of the STS map at the sample bias of -1.0 V. The white hexagon represents the CDW Brillouin zone. The six bright spots enclosed by the red circles indicate the CDW wavevectors of monolayer 1T-$NbSe_2$. **c,** STS map of monolayer 1T-$NbSe_2$ recorded at the sample bias of 0.2 V with the same location as panel a. **d,** FFT image of panel c. The spots enclosed by the yellow circles are related to the charge modulation with the wavelength larger than $\sqrt{3}$ times of the CDW wavevectors. **e,** STS map of graphene substrate recorded at the sample bias of 0.2 V. **f,** FFT image of panel e. The outer (inner) six bright spots enclosed by the green (orange) circles indicate the graphene lattice (graphene-SiC moiré superlattice).

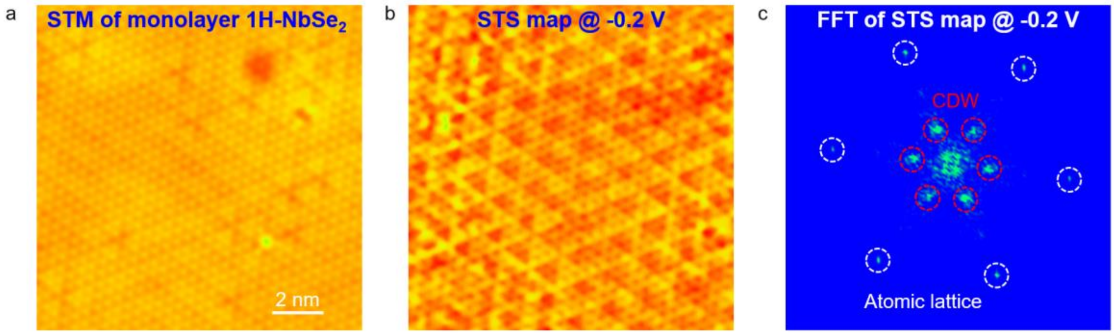


**Supplementary Fig. 9 | Electronic properties of monolayer 1H-$NbSe_2$. a,** STM image of monolayer 1H-$NbSe_2$. **b,** Corresponding STS map recorded at the sample bias of -0.2 V. **c,** Corresponding FFT images of the STS map. The outer (inner) six bright spots enclosed by the white (red) circles indicate the $1 \times 1$ atomic ($3 \times 3$ CDW) wavevectors of monolayer 1H-$NbSe_2$. There is no additional long-range charge modulation with the wavelength larger than the CDW wavevectors, indicating the absence of spinon excitations.

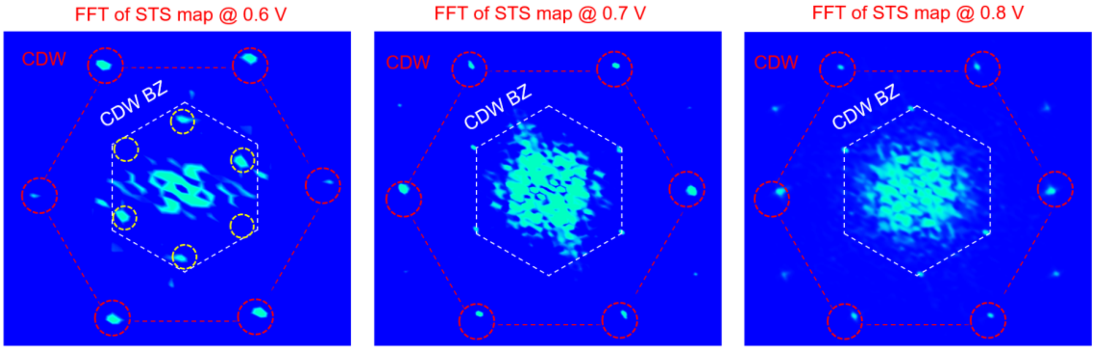


**Supplementary Fig. 10 | FFT images of the STS maps at the sample bias of 0.6 V, 0.7 V, and 0.8 V, from left to right, respectively.** The six bright spots enclosed by the red circles indicate the CDW wavevectors of monolayer 1T-$NbSe_2$, while the spots enclosed by the yellow circles are related to the charge modulation with their real-space wavelength being √3 times larger (correspondingly smaller in momentum space) of the CDW wavevectors. The white hexagon represents the CDW Brillouin zone (BZ).

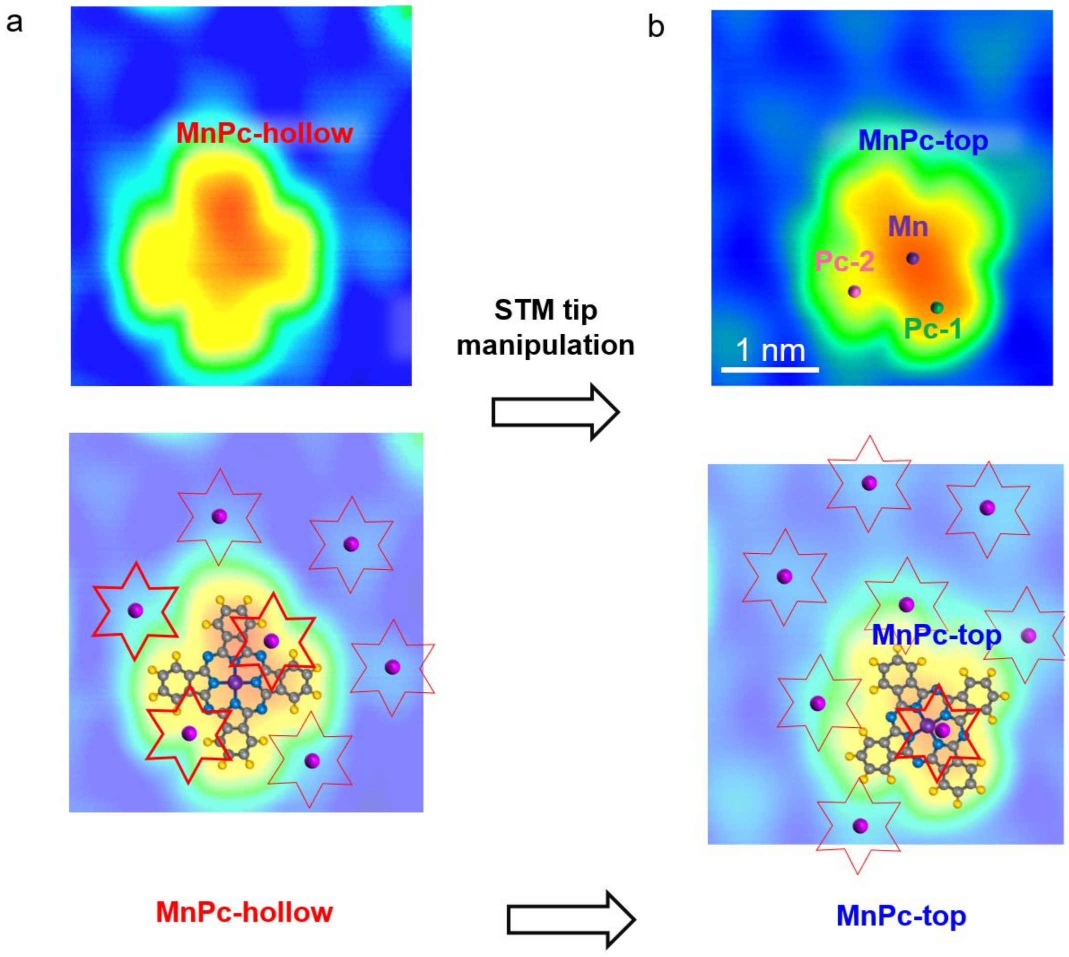


**Supplementary Fig. 11 | MnPc molecules with two topographic configurations via an STM tip manipulation technique. a,** Topographic STM image of MnPc molecules adsorbed off the SOD motifs. **b,** After the STM tip manipulation, the molecule adsorbed on the SOD motifs. The four lobes of the MnPc molecules are roughly classified into Pc-1 and Pc-2, given the lobe locating on and off a SOD motif, respectively.

## Supplementary Note 2: Spatial variations of the resonance peak near the UHB edge.

Supplementary Fig. 12 shows a typical STM image of the MnPc-top configuration and the corresponding STS map at the resonance peak energy near the UHB edge, where the center Mn ion locates on a SOD motif, most of the right-Pc locates on a SOD motif, and the up-, bottom-, and left-Pc locate off SOD motifs. From Supplementary Fig. 12, we can find out that the resonance peak near the UHB edge of the MnPc-top configuration mainly locates at the center Mn ion and the right-Pc positions, with the intensity ratio between the center Mn and the up-Pc of about 2 (It's worth noting that STS mappings can only reflect the relative intensities at specific energies). Such a result implies that the overlap between the holon state in the QSL monolayer 1T-$NbSe_2$ and the magnetic moment of the Mn/right-Pc position is larger than that of the other three Pc positions.

Supplementary Fig. 13 shows a typical STM image of the MnPc-hollow configuration and the corresponding STS map at the resonance peak energy near the UHB edge, where the both the center Mn ion and the four Pc positions locate off SOD motifs. From Supplementary Fig. 13, we can find out that the resonance peak near the UHB edge of the MnPc-top configuration mainly locates at the center Mn ion position, with the peak intensity ratio between the center Mn and the up-left-Pc of about 4, implying the dominate overlap between the holon state in the QSL monolayer 1T-$NbSe_2$ and the magnetic moment of the Mn ion.

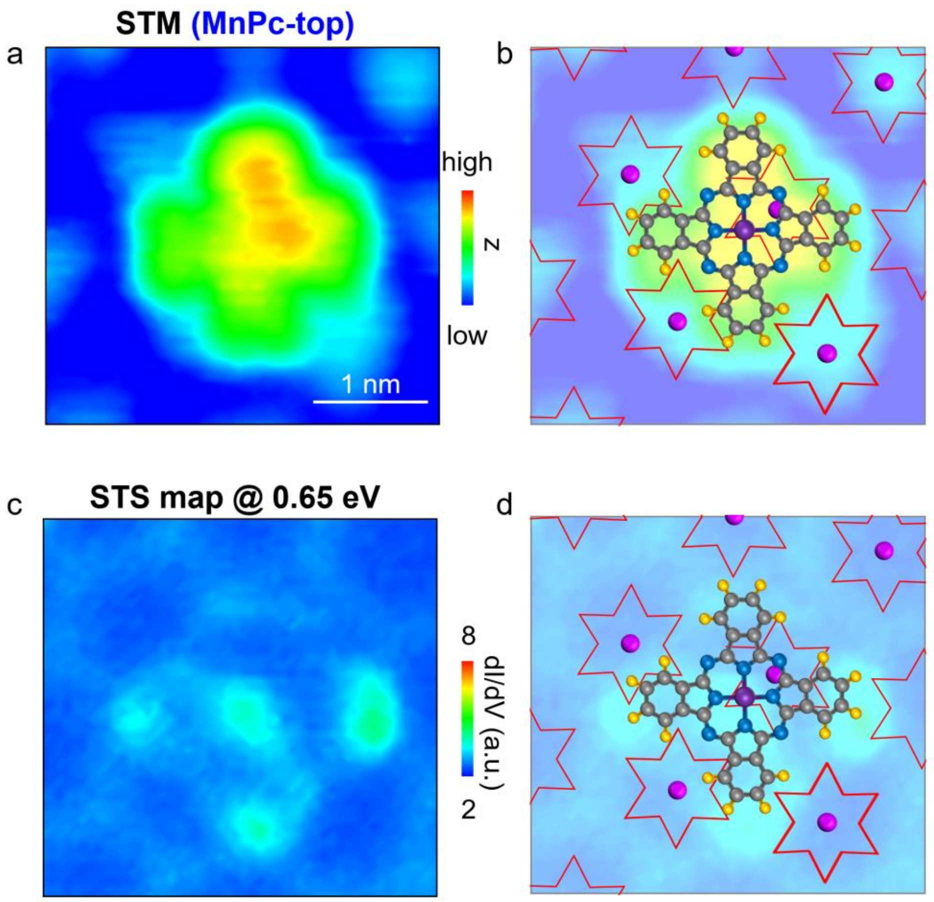


**Supplementary Fig. 12 | Spatially resolved intensities of the resonance peak appearing near the UHB edge for the MnPc-top configuration. a,b,** Topographic STM image of a MnPc molecule adsorbed on a SOD motif. **c,d,** Corresponding STS map at the resonance peak energy of 0.65 eV, reflecting the spatial variations of the spinon Kondo effect.

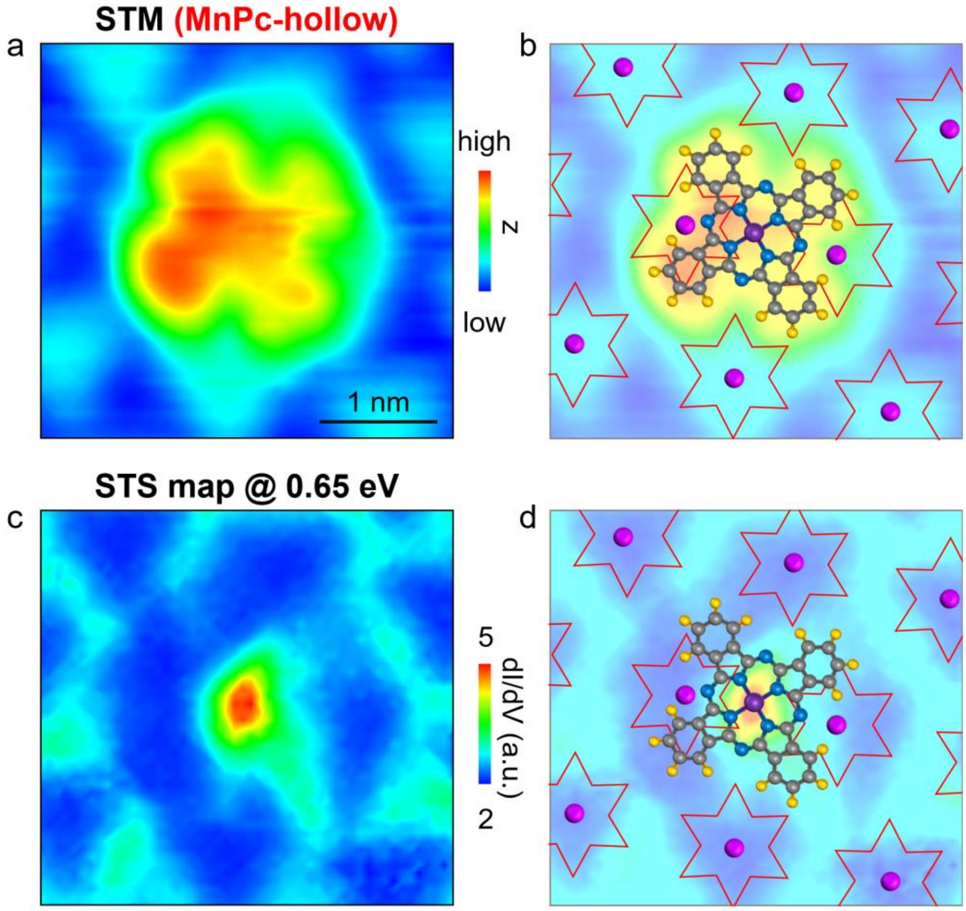


**Supplementary Fig. 13 | Spatially resolved intensities of the resonance peak appearing near the UHB edge for the MnPc-hollow configuration. a,b,** Topographic STM image of a MnPc molecule adsorbed off a SOD motif. **c,d,** Corresponding STS map at the resonance peak energy of 0.65 eV, reflecting the spatial variations of the spinon Kondo effect.

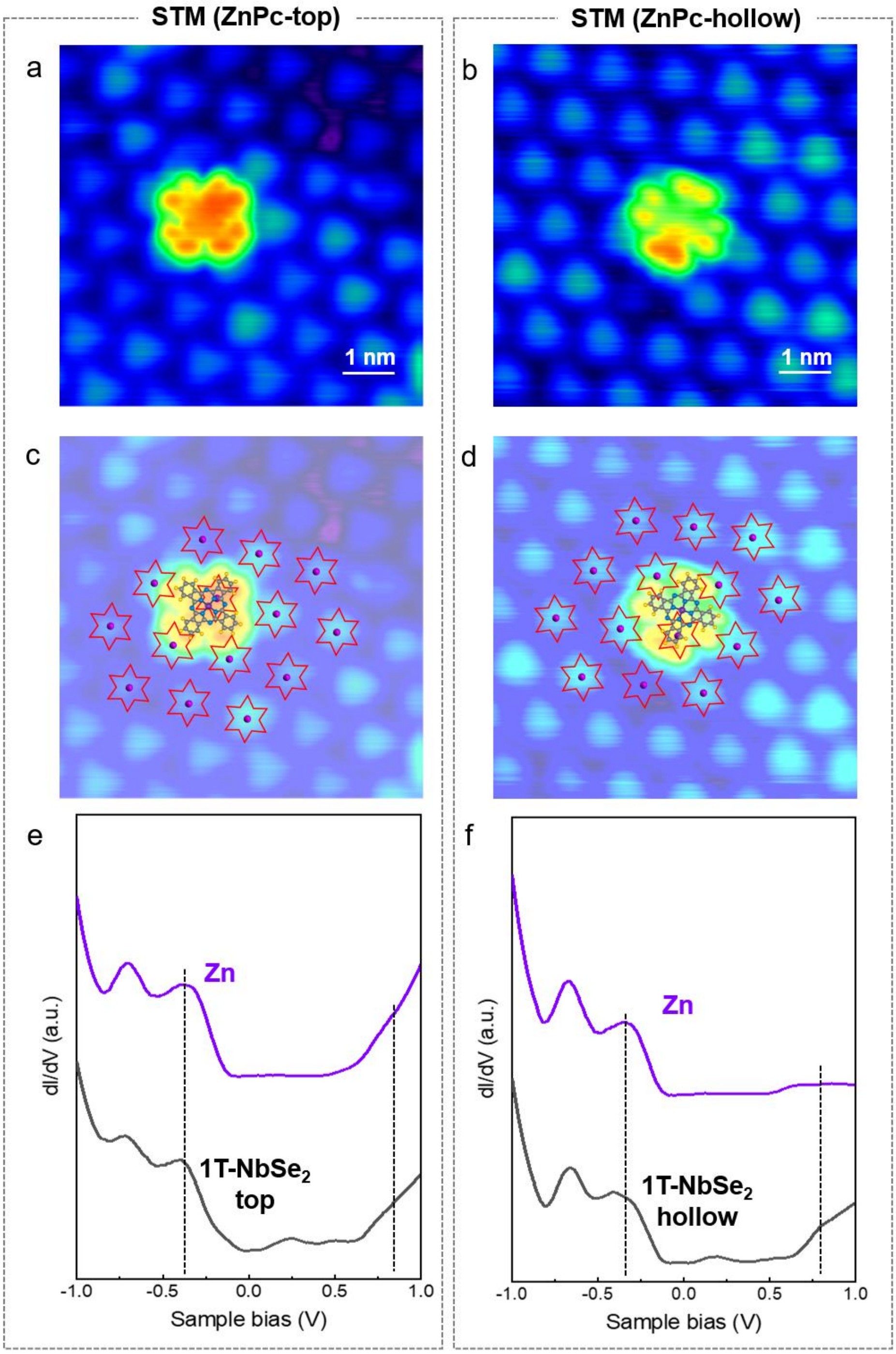


**Supplementary Fig. 14 | Atomic and electronic structures of a ZnPc molecule on monolayer 1T-NbSe$_2$. a-d,** STM images and atomic structures of a ZnPc molecule on monolayer 1T-NbSe$_2$, with the central Zn atom on the top and hollow sites of a SOD motif, respectively. **e,f,** STS spectra recorded on pristine monolayer 1T-NbSe$_2$ as well as on Zn atom of the ZnPc-top and ZnPc-hollow. No additional resonance peaks appear at the band edges, implying the absence of the spinon Kondo effect.

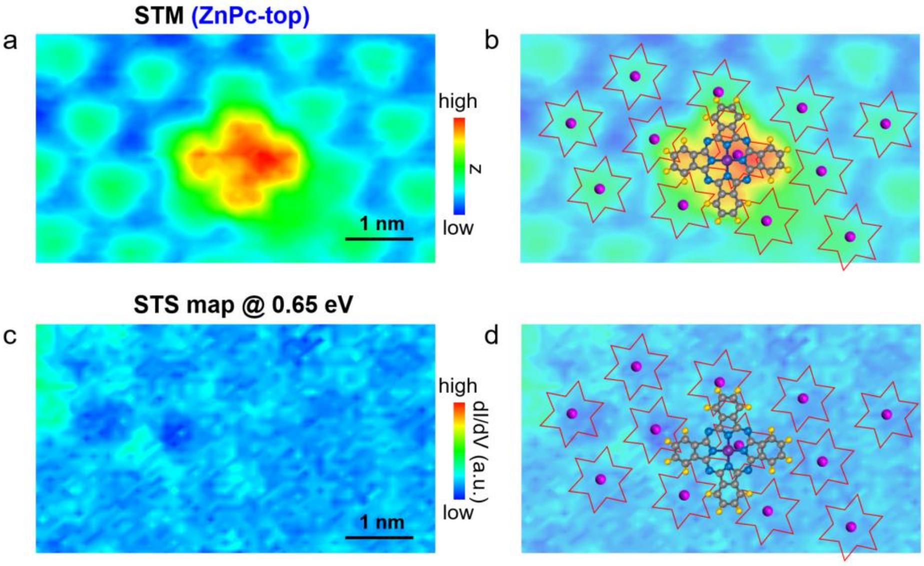


**Supplementary Fig. 15 | Spatially resolved charge intensities near the UHB edge for the ZnPc-top configuration. a,b,** Topographic STM image of an individual non-magnetic ZnPc molecule adsorbed on a SOD motif. **c,d,** Corresponding STS map at the resonance peak energy of 0.65 eV, reflecting the absence of the spinon Kondo effect.

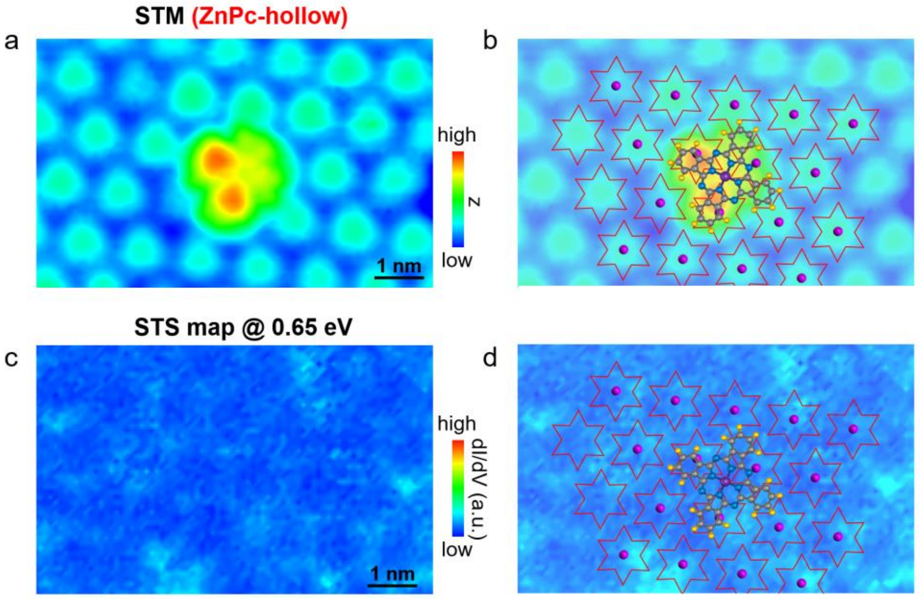


**Supplementary Fig. 16 | Spatially resolved charge intensities near the UHB edge for the ZnPc-hollow configuration. a,b,** Topographic STM image of an individual non-magnetic ZnPc molecule adsorbed off a SOD motif. **c,d,** Corresponding STS map at the resonance peak energy of 0.65 eV, reflecting the absence of the spinon Kondo effect.

## Supplementary Note 3: Spinon Kondo effect

The MnPc molecule with $S = 3/2$ is treated as an idealized local magnetic impurity in the theoretical calculation. In the underscreened Kondo regime, a local spin of $S = 1$ is expected to remain in the magnetic impurity, while the spin $S = 1/2$ undergoes Kondo spin exchange with the itinerant spinons[1]. For the magnetic impurity deposited on the U(1) quantum spin liquid (QSL) with spinon Fermi surface (SFS), the magnetic impurity couples to the QSL electronic states individually in the spin and charge channels. In the slave rotor mean field description, the Matsubara Green's functions for the auxiliary fermion and the charged boson at the impurity take the form

$$G_{a,\sigma}(i\omega_n) = \frac{1}{i\omega_n - \epsilon_0 + h - w^2 G_{f,\sigma}(i\omega_n, \boldsymbol{R}, \boldsymbol{R})},$$

$$G_{X_d}(i\nu_n) = \frac{1}{\frac{\nu_n^2}{U} - \frac{2i\nu_n h}{U} + \lambda - u^2 G_X(i\nu_n, \boldsymbol{R}, \boldsymbol{R})},$$

with the imaginary-time Green's function $G_{f,\sigma}(i\omega_n, \boldsymbol{R}, \boldsymbol{R}) = \sum_{\boldsymbol{k}} \frac{1}{i\omega_n - \xi_{\boldsymbol{k}}}$ for the spinon and $G_X(i\nu_n, \boldsymbol{R}, \boldsymbol{R}) = \sum_{\boldsymbol{k}} \left(\frac{1}{i\nu_n + \epsilon_{\boldsymbol{k}}} - \frac{1}{i\nu_n - \epsilon_{\boldsymbol{k}}}\right)$ for the chargon, respectively. Here the coupling fields $w$, $u$, and the Lagrangian multipliers $\lambda$, $h$ take the value at the saddle point with the minimal free energy, based on the mean-field theory. $\epsilon_0$ is the impurity on-site energy, $U$ is the onsite repulsion, and $\sigma = \uparrow / \downarrow$ is the spin index. $\omega_n$ and $\nu_n$ are the fermionic and bosonic Matsubara frequencies, respectively. The spinon and chargon states in the U(1) QSL with SFS have the Matsubara Green's function as

$$G^0_{f,\sigma}(i\omega_n, \boldsymbol{R}, \boldsymbol{R}) = \frac{1}{G^{-1}_{f,\sigma}(i\omega_n, \boldsymbol{R}, \boldsymbol{R}) - \frac{w^2}{i\omega_n - \epsilon_0 + h}},$$

$$G^0_X(i\nu_n, \boldsymbol{R}, \boldsymbol{R}) = \frac{1}{G_X^{-1}(i\nu_n, \boldsymbol{R}, \boldsymbol{R}) - \frac{u^2}{\frac{\nu_n^2}{U} - \frac{2i\nu_n h}{U} + \lambda}}.$$

Given the mean field parameters $w = -0.03$ eV, $u = 0.16$ eV, $h = 0.002$ eV, $\lambda = 1.28$ eV, and the onsite repulsion $U = 5$ eV at the impurity, we obtain the auxiliary fermion density of states (DOS)

$$\rho^0_{a,\sigma}(\omega) = -\frac{1}{\pi}\mathrm{Im}G^0_{a,\sigma}(i\omega_n \to \omega + i0^+),$$

the charged boson DOS

$$\rho^0_{X_d}(\omega) = \left|-\frac{1}{\pi}\mathrm{Im}G_{X_d}(i\nu_n \to \omega + i0^+)\right|,$$

the QSL spinon DOS

$$\rho^0_{f,\sigma}(\omega) = -\frac{1}{\pi}\mathrm{Im}G^0_{f,\sigma}(i\omega_n \to \omega + i0^+),$$

and the QSL chargon DOS

$$\rho^0_{X}(\omega) = \left|-\frac{1}{\pi}\mathrm{Im}G_{X}(i\nu_n \to \omega + i0^+)\right|.$$

The Matsubara Green's functions for the electronic state at the impurity and QSL electronic states are obtained through the convolution

$$G^0_{d,\sigma}(i\omega_n) = \frac{1}{\beta}\sum_{\nu_n} G_{a,\sigma}(i\omega_n + i\nu_n)G_{X_d}(i\nu_n),$$

$$G_{c,\sigma}(i\omega_n, \boldsymbol{R}, \boldsymbol{R}) = \frac{1}{\beta}\sum_{\nu_n} G^0_{f,\sigma}(i\omega_n + i\nu_n, \boldsymbol{R}, \boldsymbol{R})G^0_X(i\nu_n, \boldsymbol{R}, \boldsymbol{R}).$$

Here $\beta^{-1} = k_b T$ is the Boltzmann constant. The corresponding DOS spectra are plotted in Supplementary Fig. 18 as well. Here the band dispersions of the spinon and chargon in the QSL are

$$\xi_{\boldsymbol{k}} = 2t_f\left(2\cos\frac{1}{2}k_x a\cos\frac{\sqrt{3}}{2}k_y a + \cos k_x a\right) - \mu_f$$

$$\epsilon_{\boldsymbol{k}} = 2t_X\left(2\cos\frac{1}{2}k_x a\cos\frac{\sqrt{3}}{2}k_y a + \cos k_x a - 3\right) + \Delta.$$

The longitudinal gauge field fluctuations in the QSL gives rise to the gauge binding interaction

$$H_{U_b} = U_b\sum_{\sigma}(f^\dagger_{\sigma,\boldsymbol{R}}f_{\sigma,\boldsymbol{R}}a^\dagger_{\boldsymbol{R}}a_{\boldsymbol{R}} - f^\dagger_{\sigma,\boldsymbol{R}}f_{\sigma,\boldsymbol{R}}b^\dagger_{\boldsymbol{R}}b_{\boldsymbol{R}}),$$

with $f_{\sigma,\boldsymbol{R}}^{(\dagger)}$, $a_{\boldsymbol{R}}^{(\dagger)}$, $b_{\boldsymbol{R}}^{(\dagger)}$ being the annihilation (creation) operators for the spinon, holon, and doublon states in the QSL. Taking the gauge binding $U_R$ into account, the Matsubara Green's function for the impurity electronic state reads

$$G_{d,\sigma}(i\omega_n) = \sum_{i,j=1,2}\left\{G_{0,\sigma}^{11}(i\omega_n) + G_{0,\sigma}^{12}(i\omega_n)U_R\sigma_z\left[1 - G_{0,\sigma}^{22}(i\omega_n)U_R\sigma_z\right]^{-1}G_{0,\sigma}^{21}(i\omega_n)\right\}_{ij},$$

with the form of $G_{0,\sigma}(i\omega_n)$ given in the method section. The local electronic DOS of the impurity is then $\rho_{d,\sigma}(\omega) = -\frac{1}{\pi}\mathrm{Im}G_{d,\sigma}(i\omega_n \to \omega + i0^+)$. As the gauge binding interaction increases, a pair of band edge resonance peaks in $\rho_{d,\sigma}(\omega)$ gradually emerge as is plotted in Supplementary Fig. 19.

In the case that the holon-spinon hole binding interaction is effectively suppressed:

$$H'_{U_b} = -U_R \sum_\sigma f_{\sigma,\boldsymbol{R}}^\dagger f_{\sigma,\boldsymbol{R}} b_{\boldsymbol{R}}^\dagger b_{\boldsymbol{R}},$$

the Matsubara Green's function for the impurity electronic state takes the form

$$G'_{d,\sigma}(i\omega_n) = \sum_{i,j=1,2}\left\{G_{0,\sigma}^{11}(i\omega_n) + G_{0,\sigma}^{12}(i\omega_n)U_R\sigma'\left[1 - G_{0,\sigma}^{22}(i\omega_n)U_R\sigma_z\right]^{-1}G_{0,\sigma}^{21}(i\omega_n)\right\}_{ij}$$

with $\sigma' = \begin{pmatrix} 0 & 0 \\ 0 & -1 \end{pmatrix}$. The corresponding impurity electronic DOS is $\rho'_{d,\sigma}(\omega) = -\frac{1}{\pi}\mathrm{Im}G'_{d,\sigma}(i\omega_n \to \omega + i0^+)$, which has only one single resonance peak at the bottom of the UHB as plotted in Fig. 4d (green line).

From the idealized local magnetic impurity model on the QSL with spinon Fermi surface, it is known that spinon Kondo effect arises from the spin exchange between the itinerant spinons and the local spin with $S$ = 1/2 at the impurity. In the case of $S$ = 3/2 spin, the local $S$ = 1/2 spin gets screened by Kondo cloud formed by the spinons, and the remaining local spin with $S$ = 1 is decoupled. In the case of $S$ = 1/2 spin, the

local spin gets fully screened by the spinon Kondo cloud. In the case of $S = 0$ spin, there is no spin exchange between the magnetic impurity and the itinerant spinons. As a result, one can see that the spinon Kondo effect for different spins differs in the Kondo screening. For the spinon Kondo resonance peaks observed in our experiment, the resonance peaks are due to the joint effect of the spinon Kondo coupling and the gauge binding effect. As long as the local spin at the impurity has finite coupling with the itinerant spinons, sufficiently large gauge binding interaction can always give rise to the band edge resonance peaks. As a result, we expect that the band edge resonance peaks appear at the magnetic impurity with $S = 1/2$ spin and $S = 3/2$ spin, but disappear if there is no local spin at the impurity.

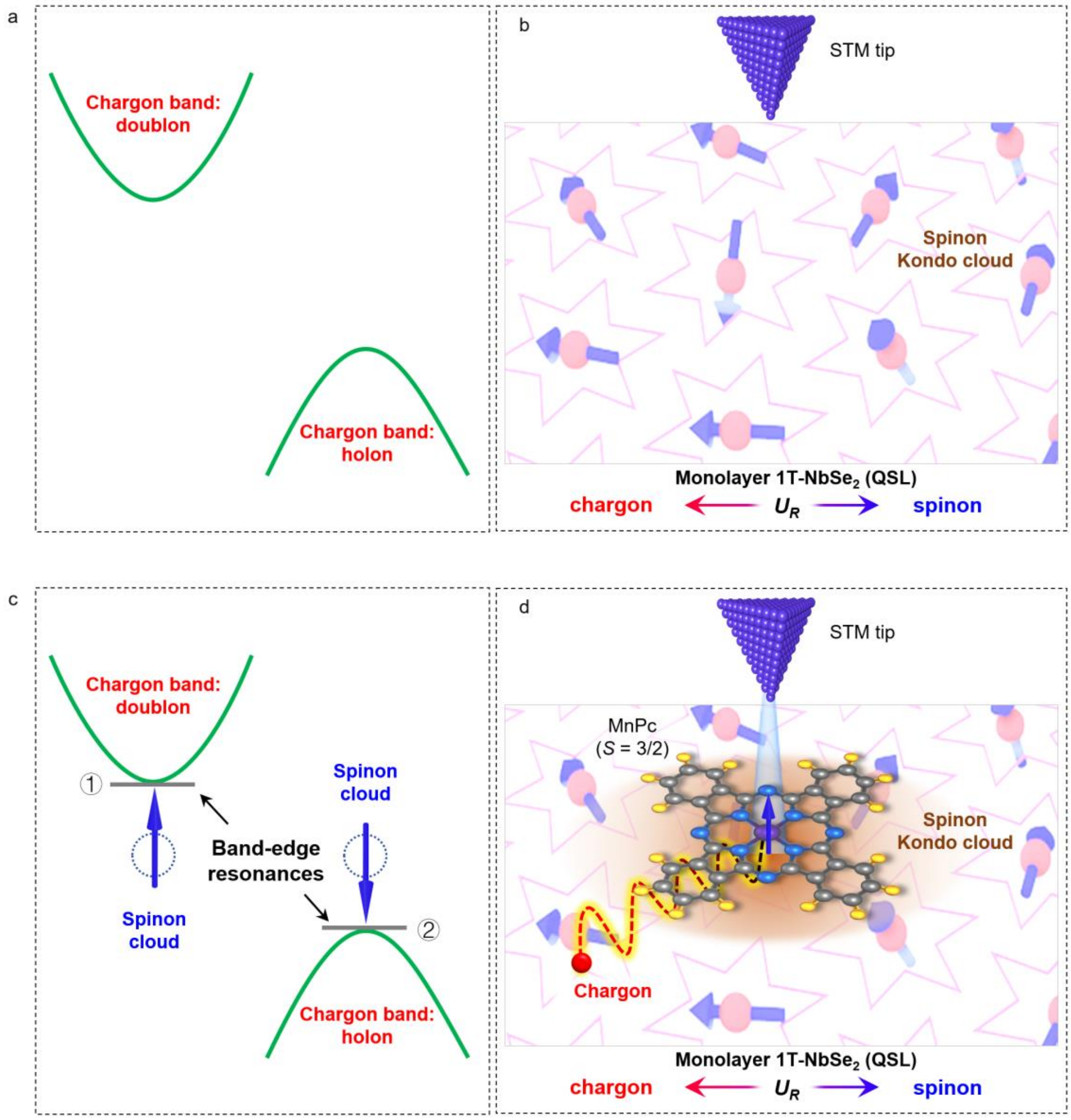


**Supplementary Fig. 17 | The DOS in the pristine QSL and at the position of the adsorbate acting like a Kondo-impurity. a,** Energy diagram of the QSL state. **b,** Schematic diagram of the QSL state detected via STM. **c,** Energy diagram of the band edge resonance states. **d,** Physical mechanism of the interaction between a QSL state and a local magnetic moment. A magnetic impurity in a QSL candidate can result in a spinon Kondo screening cloud (brown shadow). Such a cloud attracts a chargon in QSL under the spin-charge binding interaction.

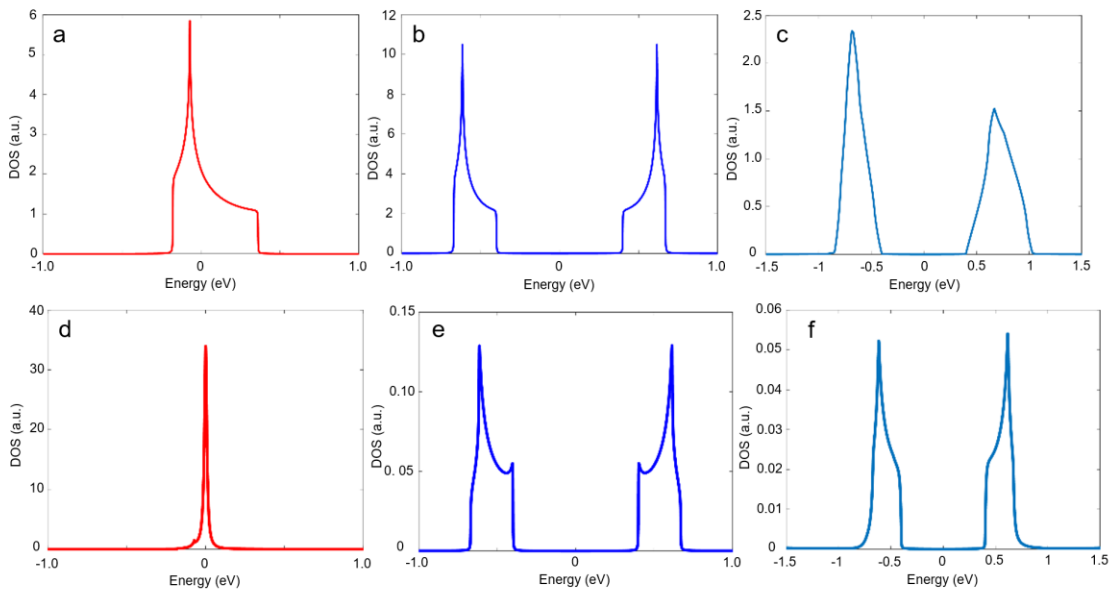


**Supplementary Fig. 18 | The DOS in the pristine QSL and at the position of the adsorbate acting like a Kondo-impurity. a-c,** The DOS of the spinon, chargon, and the electronic states in the pristine QSL, respectively. In the case of the pristine QSL, there is no magnetic impurity present. The DOS can be obtained from the Green's functions through setting the coupling parameters $u$=$w$=0 eV. **d-f,** The DOS of the auxiliary fermion, charged boson and electronic state at the adsorbate position acting like an impurity, respectively. Here, the spin exchange between the impurity and the QSL gives rise to the spinon Kondo resonance in the spin channel.

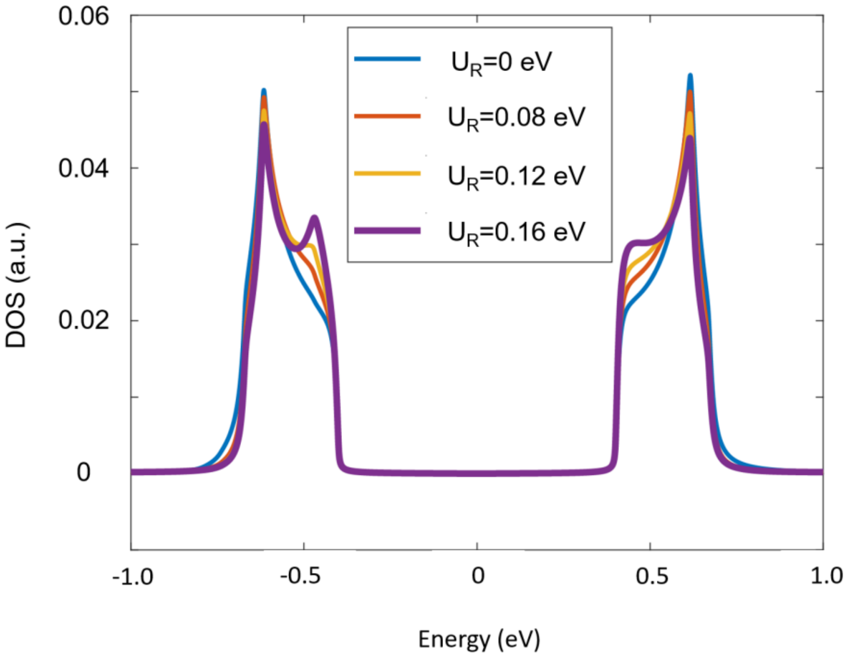


**Supplementary Fig. 19 | The emergence of a pair of band edge resonance peaks in the local electronic DOS of the local magnetic impurity.** The gauge binding is considered in both the doublon-spinon channel and the holon-spinon hole channel. The spinon-chargon binding interaction $U_R$ modifies the impurity electronic Green's function, which is expected to induce the spectral weight to transfer from the bulk Hubbard bands to the band edges. Experimentally, $U_R$ depends on the location of a magnetic molecular related to the SOD motif.

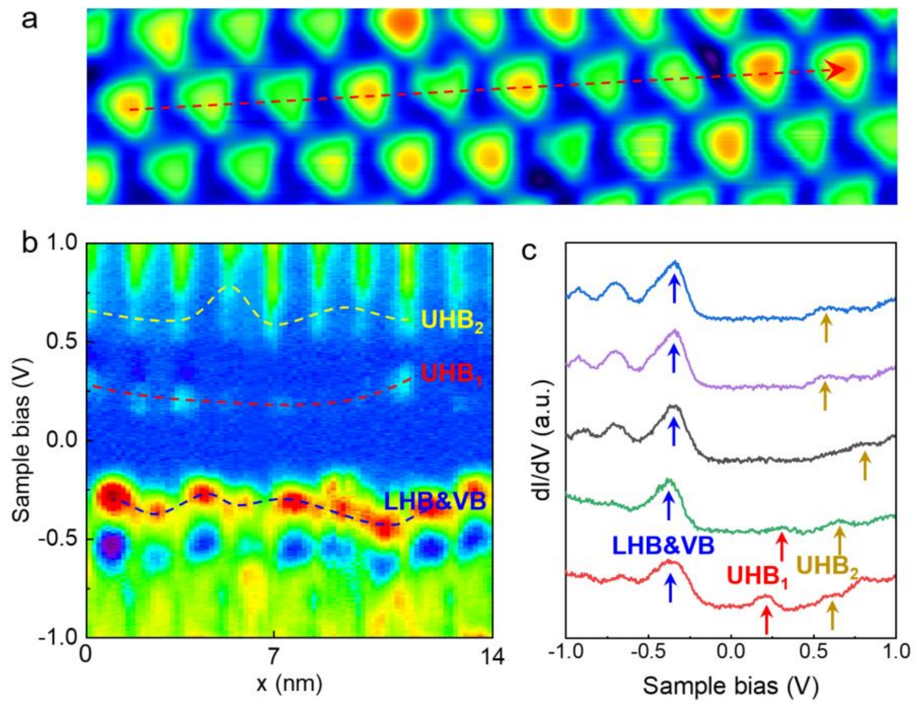


**Supplementary Fig. 20 | Spatially resolved STS spectra of heterogeneous monolayer 1T-NbSe$_2$. a,** Typical STM image of monolayer 1T-NbSe$_2$ with atomic defects, exhibiting enhanced or weakened SOD spots. **b,** Spatially resolved STS spectra recorded along the red arrow marked in panel a. The energies and intensities of LHB & VB, UHB$_1$, and UHB$_2$ are strongly changed, as marked by the blue, red, and yellow dashed lines, respectively. **c,** Typical STS spectra extracted from panel b. The UHB$_1$ and UHB$_2$ can shift in energy or even vanish at different locations.

## Supplementary Note 4: Differences in electronic properties among 1T-$NbSe_2$, 1T-$TaS_2$, and 1T-$TaSe_2$.

The QSL behavior in monolayer 1T-$NbSe_2$ should not be directly viewed as a simple extrapolation from 1T-$TaSe_2$ to 1T-$NbSe_2$ by intuition, but needs to be carefully investigated, because there are still many differences in electronic properties among 1T-$NbSe_2$, 1T-$TaS_2$, and 1T-$TaSe_2$, as summarized in Supplementary Tab. 1. For example, for the bilayer system, bilayer 1T-$NbSe_2$ is usually a metal, while bilayer 1T-$TaSe_2$ is still a Mott insulator with a reduced energy gap. No experimental results have been reported on the electronic structures of bilayer 1T-$TaS_2$. For the surface states of the bulk, 1T-$TaSe_2$ and 1T-$TaS_2$ can be an insulator or a metal, dependent on the interlayer stacking. However, bulk 1T-$NbSe_2$ has not been successfully synthesized up till now. Since the bilayer and bulk samples of 1T-$NbSe_2$, 1T-$TaS_2$, and 1T-$TaSe_2$ exhibit completely different properties, the QSL state in monolayer 1T-$NbSe_2$ cannot be deduced only by the correlated insulating behaviors before the report of our experimental results.

**Supplementary Table 1 | Summarization of discrepancies among 1T-$NbSe_2$, 1T-$TaS_2$, and 1T-$TaSe_2$.**

| | 1T-$NbSe_2$ | 1T-$TaS_2$ | 1T-$TaSe_2$ |
|---|---|---|---|
| Bi-layer | Metal<br>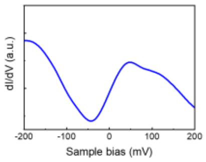<br>[this work] | Not reported | Mott insulator<br>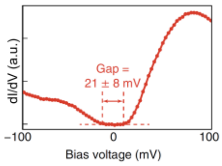<br>[*Nat. Phys.* 17, 1154 (2021)] |
| Surface states for bulk | Not reported | Metal/insulator (Stacking dependent)<br>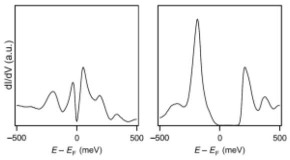<br>[*Nat. Commun.* 11, 2477 (2020)] | Metal/insulator (Stacking dependent)<br>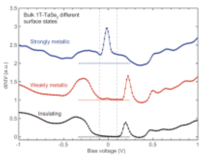<br>[*Phys. Rev. B* 106, 075153 (2022)] |
| QSL in mono-layer | U(1) QSL<br>[this work] | Not reported | U(1) QSL<br>[*Nat. Phys.* 17, 1154 (2021)] |
| QSL in bulk | Not reported | $Z_2$ QSL<br>[*npj Quantum Mater.* 6, 69 (2021)] | Not reported |
| Spinon Kondo effect | Monolayer, molecule with $S$ = 3/2<br>[this work] | Not reported | Monolayer, atom with $S$ = 1/2<br>[*Nat. Phys.* 18, 1335 (2022)] |

## References


1. Nagaosa, N. Quantum Field Theory in Strongly Correlated Electronic Systems (Springer, 1999).
